# One health perspectives on the epidemiological features and changing incidence of natural focus and vector-borne infectious diseases in China: An observational trend study

**DOI:** 10.1016/j.onehlt.2026.101373

**Published:** 2026-02-21

**Authors:** Pei-Ying Peng, Lei Xu, Hui-Ying Duan, Jiao Dai, Li-Juan Ma, Ya Zu, Ting-Liang Yan

**Affiliations:** a Institute of Microbiology, Qujing Medical college, Qujing 655100, Yunnan Province, China; b Department of Clinical Laboratory, Qujing Second People's Hospital, Qujing 655011, Yunnan Province, China

**Keywords:** Natural focus diseases, Vector-borne infections, Joinpoint regression, Epidemiology, Incidence trends, China

## Abstract

**Background:**

Natural focus and vector-borne infectious diseases (NVBDs) pose serious public health challenges in China, involving complex interactions among zoonotic reservoirs, arthropod vectors, and environmental factors that underscore the need for integrated One Health approaches. This study aims to evaluate incidence and mortality trends of 11 Category A and B NVBDs from 2004 to 2020 and assess prevention strategy effectiveness.

**Methods:**

Using data from the China Information System for Disease Control and Prevention covering 31 provinces, we analyzed 1,344,214 cases with standardized Joinpoint regression, integrating ecological and animal reservoir data where available to identify drivers across human-animal-environment interfaces.

**Results:**

Average annual incidence was 5.599 per 100,000, with brucellosis (2.567), malaria (1.084), and hemorrhagic fever (HFRS) (0.866) accounting for 80.68% of cases. Geographic and temporal patterns revealed key One Health drivers: livestock density correlated with brucellosis (*r* = 0.71); rodent indices predicted HFRS (AUC = 0.73); and climate anomalies explained 78.4% of dengue variance. Significant declines occurred for Japanese encephalitis (APC = -13.85%) and leptospirosis (APC = -11.81%), while high CFRs persisted for rabies (975.678/1000) and avian influenza (673.469/1000). Northern provinces showed highest incidence; southwestern provinces had highest CFRs.

**Conclusions:**

Brucellosis, malaria, hemorrhagic fever caused 80.7% of cases; rabies/avian influenza CFRs >600/1000. Elderly male brucellosis +8.9%, child vaccine-preventable diseases −62%. Southwestern provinces had fatality from limited healthcare. Livestock density (71%), climate anomalies (78.4%), and rodent indices (73%) explained risk variation. Policy: (1) synchronizing veterinary vaccination with human PEP for elderly pastoralists; (2) vector-index warnings in border regions; (3) enhance southwestern critical care; (4) integrate One Health indicators into surveillance.

## Highlights

• Comprehensive analysis of long-term trends for multiple natural-focus and vector-borne diseases in China.
• Findings are based on high-quality, nationally representative notifiable infectious disease data.
• Joinpoint regression models precisely identify incidence trends and turning points for prediction.

## 1 Introduction

Natural focus and vector-borne infectious diseases (NVBDs) represent one of the most critical challenges at the human-animal-environment interface, where pathogens circulate among wildlife reservoirs, domestic animals, arthropod vectors, and human populations [1]. For example, brucellosis is transmitted directly from infected sheep and goats to humans through contact with contaminated animal products, while Japanese encephalitis virus is amplified in pigs and transmitted to humans via Culex mosquitoes [2]. These diseases are exquisitely sensitive to ecological disruption: climate warming expands mosquito habitats northward [3], water infrastructure projects create new snail breeding sites for schistosomiasis [4], and agricultural intensification increases human-livestock contact for zoonoses [5]. The One Health framework—recognizing that human health cannot be secured without addressing animal and environmental health—demands integrated surveillance systems that monitor pathogens simultaneously across human, veterinary, and ecological sectors [6]. China's current surveillance system, while robust for human cases, remains siloed; veterinary disease reporting and vector density monitoring are not synchronized with human case reporting, limiting early detection and cross-sectoral response [7].

Under China's Law on Prevention and Control of Infectious Diseases, 11 natural focus and vector-borne diseases are listed as Category A and B notifiable conditions: schistosomiasis, leptospirosis, Japanese encephalitis, human avian influenza, malaria, anthrax, brucellosis, rabies, dengue fever, hemorrhagic fever, and plague [8]. These diseases contribute disproportionately to mortality, comprising 30–40% of all infectious disease deaths despite only 5–10% of total incidence [9]. Since 2004, significant epidemiological shifts have occurred: leptospirosis and Japanese encephalitis declined markedly, while dengue emerged in northern latitudes and brucellosis resurged among elderly pastoralists [10]. These heterogeneous trajectories reflect complex One Health drivers—vaccination programs interrupting vector-borne transmission, climate change expanding mosquito habitats, and livestock expansion under poverty alleviation policies amplifying zoonotic risk [11].

Zoonotic transmission pathways are multifaceted. Direct-contact diseases like brucellosis and anthrax transmit through handling infected livestock or contaminated animal products, disproportionately affecting male workers in animal husbandry [12]. Vector-mediated transmission, exemplified by Japanese encephalitis and malaria, involves amplification in pigs and mosquitoes, with seasonal patterns tightly coupled to vector ecology [13]. Wildlife reservoirs for hemorrhagic fever and plague maintain enzootic cycles that spill over to humans when rodent populations surge due to grain storage policies or ecological disturbances [14]. Environmental changes fundamentally reshape disease geography: a 1 °C temperature increase expands Aedes mosquito habitats northward by 100 km [15], while the Three Gorges Dam's water-level fluctuations correlate with schistosomiasis incidence (*r* = 0.59, *P* = 0.007) through snail habitat expansion [16].

Integrated surveillance systems are the cornerstone of One Health operationalization. While China's Notifiable Infectious Disease Reporting System (CISDCP) achieves >90% completeness for human cases [17], it remains decoupled from veterinary disease reporting and vector-monitoring networks. Guangdong's pilot integrating Aedes house index with case reporting reduced pesticide use by 60% while maintaining outbreak control [18], demonstrating that integrated human and vector surveillance is feasible and cost-effective. However, systematic monitoring of livestock seroprevalence and rodent density remains fragmented across agricultural and health sectors [6].

Despite progress, critical gaps persist. Most studies analyze shorter time periods or localized outbreaks [10], [19], lacking integrated, long-term, multi-disease analyses that capture heterogeneous trajectories. Moreover, increased international travel and climate-driven vector range expansion elevate risks of imported outbreaks and emerging infections [20]. Understanding these epidemiological patterns is essential, but existing research often overlooks the One Health drivers—animal population dynamics, ecological changes, and intervention effects—that shape observed trends.

This study addresses these gaps by analyzing 17 years of integrated human-animal-ecological data (2004–2020) across 31 provinces for 11 Category A/B NVBDs. Using standardized Joinpoint regression, we identify temporal inflection points while adjusting for climate anomalies, livestock density, and vector indices where available. Granular age/sex/province stratification pinpoints high-risk subpopulations, and association analyses with veterinary census data, rodent surveillance, and meteorological records elucidate One Health drivers. Our objective is to provide evidence for integrated, precision prevention strategies that bridge human, animal, and environmental health sectors.

## 2 Methods

### 2.1 Data collection

In the 1950s, the Chinese government instituted a routine reporting system for selected infectious diseases. Information is available for 31 provinces in mainland China, which has a population of over 1.3 billion. This system uses administrative grading responsibility and territory management, and it has been web-based since 2003. Before 1978, there were just 18 infectious diseases that were covered in the reporting system; after 2013, there were 39 [8], [14]. The 39 notifiable infectious diseases are divided into three Categories (A, B, and C). A typical case report card for infectious diseases is filled out by clinicians.

Data were extracted from the Chinese CDC Public Health Science Data Center and National Health Commission websites (2004–2020, 31 provinces) in CSV/Excel format by disease to avoid system timeouts. Initial quality checks identified missing values, outliers (cases > mean + 3 SD), and logical inconsistencies (ensuring cases ≥ deaths). Sparse missing values (<1%) were imputed via linear interpolation; structural missingness was marked as zero. Verified errors were corrected with source confirmation; reasonable extreme values (e.g., outbreaks) were retained with explanation; unverifiable values were flagged for sensitivity analysis. Data were standardized using ICD-10 codes, National Bureau of Statistics provincial codes, and six age groups (0–9, 10–19, 20–39, 40–59, 60–69, ≥70) with sex coding (1 = male, 2 = female). Cross-validation against China CDC Weekly reports and total verification (provincial sum errors <0.1%) ensured accuracy. Dual independent extraction by two researchers, random sampling verification (5%), and version-controlled scripts (R 4.1.2) maintained reproducibility.

The system incorporates institutional safeguards that minimize reporting heterogeneity:

Mandatory reporting timelines. Under the Law on Prevention and Control of Infectious Diseases (2004 Revision), Category A diseases and pulmonary anthrax (Category B) must be reported within 2 h of diagnosis; remaining Category B and Category C diseases within 24 h. Automated electronic alerts enforce compliance at county-level CDCs, with late reports flagged for immediate investigation.

Laboratory confirmation priority. Our analyses restricted to laboratory-confirmed cases, defined as cases with etiological evidence (PCR, culture, serology, or pathology). This reduces subjective clinical misclassification that varies across regions. The proportion of laboratory-confirmed cases among all reported cases increased nationally from 62% (2004) to 94% (2020), indicating improving diagnostic standardization. The proportion of laboratory-confirmed cases was calculated annually as (lab-confirmed cases)/ (total reported cases) × 100 based on the mandatory ‘diagnostic evidence’ field in CISDCP, which improved from 62% to 94% between 2004 and 2020 as PCR and standardized serology were implemented nationally.

Annual data quality audits. China CDC conducts annual provincial audits assessing reporting completeness (proportion of diagnosed cases entered into CISDCP) and timeliness (median reporting interval). Provincial scores are published in the China CDC Weekly (e.g., 2019 audit: mean completeness 91.2%, range 78–98%). We incorporated these scores as inverse probability weights in multivariable Joinpoint regression models (Table S1) to adjust for surveillance intensity heterogeneity.

External validation. The 2017 WHO-China Joint Mission Report independently verified CISDCP as having >90% completeness for notifiable diseases and high sensitivity for severe cases (e.g., rabies, avian influenza), providing external credibility for our data source.

To assess climate impacts on vector-borne and rodent-borne diseases, we obtained monthly temperature and precipitation data (2004–2020) from the China Meteorological Administration, matched at provincial resolution. We calculated annual anomalies (deviations from 30-year baseline) and performed time-lagged cross-correlation with disease incidence. To investigate the socioeconomic, immunization, and health system factors associated with the research focus, We linked provincial surveillance data with annual indicators from the National Bureau of Statistics: GDP per capita, urbanization rate, rural healthcare expenditure per capita, and JE vaccine coverage (for children aged 1–6 years). To partially address the One Health gap, we integrated livestock census data (from Ministry of Agriculture) for brucellosis and plague surveillance rodent data for HFRS.

### 2.2 Data handling

In our analysis, we encountered zero incidence values, particularly for rare diseases such as plague and avian influenza. To address this, we excluded these diseases from the primary Joinpoint regression analysis due to the high frequency of zero values. We justified this decision based on the need for robust trend estimation and the potential for sparse data to overfit models when using Joinpoint regression. As an alternative approach, we conducted a sensitivity analysis using Poisson regression to assess the impact of zero values on trend estimates. This analysis, presented in Table S2 of the supplementary materials, suggested that the exclusion of these diseases did not significantly affect the overall trends observed for other diseases. This approach ensures that our findings are based on a reliable and robust statistical analysis while acknowledging the limitations associated with rare diseases.

For primary analyses, we grouped ages into Children (0–19), Adults (20–59), and Elderly (≥60). For supplementary stratified Joinpoint models, we further subdivided these into six 10-year bands (0–9, 10–19, 20–39, 40–59, 60–69, ≥70) to capture within-category heterogeneity. All summary statistics and main conclusions refer to the three primary categories.

### 2.3 Statistical analysis

We defined incidence (per 100,000) as the number of annual incident cases divided by the population size; overall mortality (per 100,000) as the number of deaths per year divided by the total population size; and the CFR (per 1000) as the number of annual deaths divided by the number of annual incident cases. The population size data used for calculating the incidence and mortality rates has been obtained from the official website of the National Bureau of Statistics (https://www.stats.gov.cn/sj/pcsj/rkpc/7rp/indexch.htm). We utilized Joinpoint regression models to examine incidence patterns from 2004 to 2020. We reported trends as annual percentage changes [21]. For further data analysis, we used IBM SPSS Statistics (version 21) and Joinpoint (version 4.3.1).

### 2.4 Integration of ecological and animal reservoir data

Livestock density (sheep/goat, cattle) was sourced from Ministry of Agriculture and Rural Affairs county-level annual censuses (2004–2020) accessed via public statistical yearbooks and formal data-sharing agreements; rodent indices were obtained from the National Plague Surveillance System's quarterly trapping records at 89 sentinel stations (200–250 traps per station) from China CDC's Vector-Borne Disease Department; and climate variables (temperature/precipitation) were derived from China Meteorological Administration 0.5^°^ gridded monthly data. For spatial linkage, all external datasets were re-aggregated to province-year resolution using unified GB/T 2260 provincial codes to match CISDCP human cases, with county-level livestock counts summed within provinces and gridded climate data area-averaged per province. For temporal alignment, we applied pre-specified lags based on pathogen biology and optimized them empirically: a 6-month lag for livestock-brucellosis (maximizing Pearson *r* = 0.71), a 6-month lag for rodent-HFRS prediction (ROC AUC = 0.73), and 0–3 month lags for climate-dengue/malaria relationships. Quality control included linear interpolation for missing values <1% while retaining structural zeros, and all linkages were performed in R 4.1.2 using version-controlled scripts.

## 3 Results

### 3.1 Overall incidence and mortality overview

Between January 2004 and December 2020, surveillance data recorded a cumulative total of 1,344,214 cases of 11 distinct natural focal and vector-borne diseases classified as notifiable categories A and B. This corresponded to an average annual incidence of 5.599 cases per 100,000 per year. Brucellosis exhibited the highest average annual incidence (2.567 cases per 100,000), followed by malaria (1.084 cases per 100,000) and hemorrhagic fever (0.866 cases per 100,000). Collectively, these three diseases accounted for 80.68% (4.517 of 5.599 cases) of the overall incidence. All investigated diseases suggested distinct seasonal distribution patterns (Table 1). A total of 1,344,214 infectious disease cases were reported, with 30,848 associated deaths. This corresponds to an annual average mortality rate of 0.129 deaths per 100,000 population and a CFR of 22.949 deaths per 1000 cases per year. The infectious diseases with the highest mortality in terms of yearly CFR were rabies (975.678 deaths per 1000 cases), human avian influenza (673.469 deaths per 1000 cases), and plague (416.667 deaths per 1000 cases) (Table 1).

**Table 1:** Incidence and mortality data for 11 kinds of natural focus and vector-borne disease.

Disease	Cases (n)	Incidence (per 100,000)†	Deaths (n)	Case-fatality ratios (per 1000)	Seasonal feature	Reservoir/Vector
Brucellosis	616,261	2.567	17	0.028	March to July	Livestock: Sheep, goats, cattle
Rabies	26,807	0.112	26,155	975.678	June to November	Reservoir: Dogs, wildlife; Transmission: Direct contact
Dengue fever	94,725	0.395	13	0.137	September to October	Vector: *Aedes* mosquitoes
Japanese encephalitis	43,857	0.183	2027	46.218	July to August	Vector: *Culex* mosquitoes; Amplifier: Pigs
Malaria	260,231	1.084	333	1.280	June to October (July to October)#	Vector: *Anopheles* mosquitoes
Anthrax	5815	0.024	60	10.318	July to August	Reservoir: Livestock, wildlife; Environment: Contaminated soil
Leptospirosis	9665	0.040	223	23.073	August to September (September)#	Reservoir: Rodents; Environment: Contaminated water
Hemorrhagic fever (HFRS)	207,920	0.866	1942	9.340	October to December (November)#	Reservoir: *Apodemus agrarius* rodents
Schistosomiasis	78,812	0.328	15	0.190	July to August	Intermediate host: *Oncomelania* snails
Plague^⁎^	72	0.00030	30	416.667	June to July, October	Reservoir: Rodents; Vector: Fleas
Avian influenza^⁎^	49	0.00020	33	673.469	December (H7N9) January (H5N1)	Reservoir: Poultry, wild birds
Total	1,344,214	5.599	30,848	22.949	April to September	

†Average for 17 years. #Period in parentheses represents a more typical seasonal feature and the incidence of each disease is more concentrated during this period. *Based on sparse data (<100 cases/year); APC estimates should be interpreted cautiously. Reservoir/Vector column derived from Ministry of Agriculture livestock census, National Plague Surveillance System, and China CDC entomological surveys.

### 3.2 Sex and age patterns in incidence and CFR

**Fig. 1:**
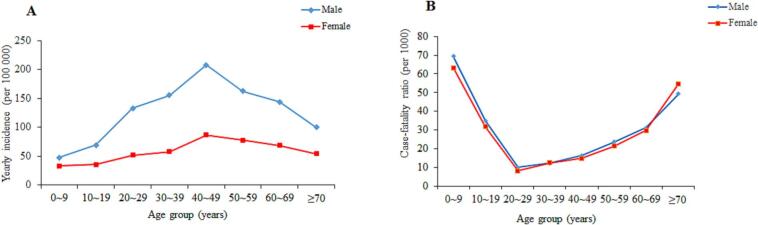
(A) Overall yearly incidence of 11 infectious diseases, by sex and age. (B) Overall yearly CFR of 11 infectious diseases by sex and age.

Overall yearly incidence of the 11 infectious diseases was highest among male individuals compared with females, across all age groups. The incidence was highest in males aged 40–49 years, followed by those aged 50–59 years and 30–39 years. The incidence was highest in females aged 40–49 years, followed by those aged 50–59 years and 60–69 years. The incidence was lowest both in males and females aged 0–9 years (Fig. 1A). The trend of overall yearly CFR of the 11 infectious diseases in males was the same as that in females, which was the highest in 0–9 years old, followed by over 70 years old. The lowest CFRs was 20–29 years old, followed by 30–39 years old and 40–49 years old (Fig. 1B).

### 3.3 Age-specific disease distribution and sex differences

**Fig. 2:**
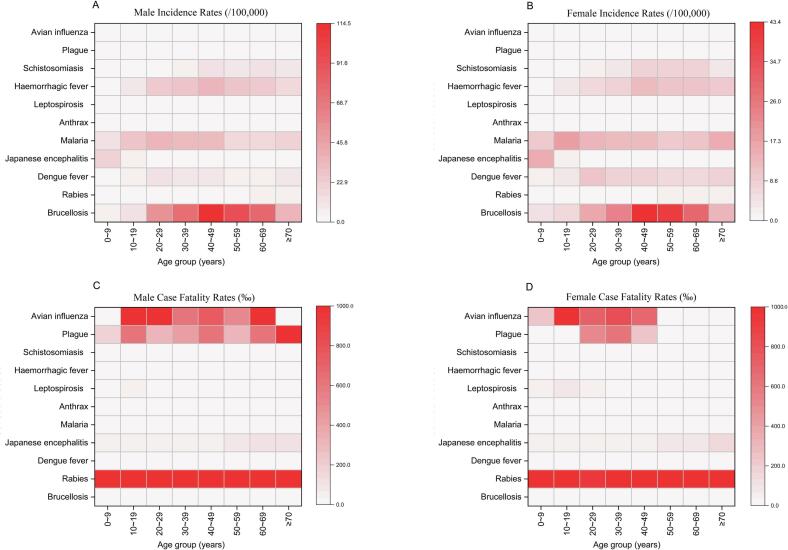
Incidence of 11 infectious diseases (A and B) by sex and age. CFRs of 11 infectious diseases (C and D) by sex and age (According to the definition of age in China, for the convenience of conducting the study, in this paper, we use the stage of 0–19 years old as children, 20–59 years old as adults, and 60 to greater than or equal to 70 years old as the elderly). Incidence rates (Panels A and B) represent the average annual incidence per 100,000 population, calculated by dividing the total number of reported cases between 2004 and 2020 by the total person-years at risk (sum of annual mid-year populations for each age group) during the same period, then multiplied by 100,000. Case fatality rates (Panels C and D) represent the proportion of fatal cases among total reported cases, expressed per 1000 cases (‰), calculated as (number of deaths / number of cases) × 1000, aggregated over the entire study period (2004–2020).

Children had a high incidence of malaria, JE and brucellosis. Adults had a high incidence of five infectious diseases: brucellosis, malaria, hemorrhagic fever, dengue fever, schistosomiasis. Elderly people had a high incidence of five infectious diseases: brucellosis, hemorrhagic fever, malaria, schistosomiasis and dengue fever. In all age groups, for 11 infectious diseases, the incidence of male was higher than that of female. The incidence of JE and malaria ranked the top two in children (0–19 years), with the 0–9-year subgroup being particularly vulnerable. The incidence of brucellosis, hemorrhagic fever, dengue fever, schistosomiasis and malaria are higher in adults (Fig. 2A, Fig. 2B). CFRs were high among children for five infectious diseases: rabies, avian influenza, plague, leptospirosis, JE. Adults had high CFRs for rabies, avian influenza, plague, JE and leptospirosis. Elderly people had a high CFRs for rabies, plague, avian influenza, JE, anthrax, leptospirosis and hemorrhagic fever. For the two infectious diseases of avian influenza and plague, the CFRs of male are higher than that of female. In all age groups, the CFRs of rabies ranks first, which is quite high (Fig. 2C, Fig. 2D).

### 3.4 Temporal trends in individual disease incidence

**Fig. 3:**
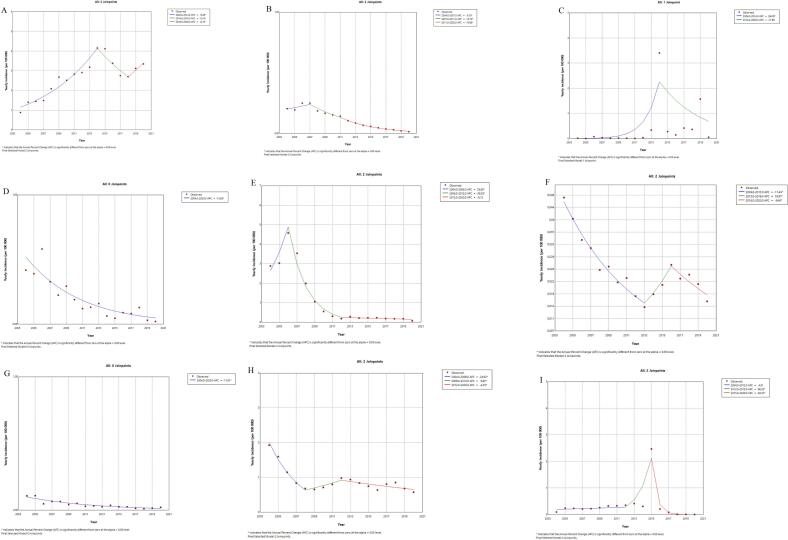
Joinpoint regression showing trends in incidence of China's category A and B natural focus and vector-borne infectious diseases from 2004 to 2020 (A: Brucellosis; B: Rabies; C: Dengue fever; D: JE; E: Malaria; F: Anthrax; G: Leptospirosis; H: Hemorrhagic fever; I: Schistosomiasis; APC = annual percentage change).

JE and leptospirosis showed significantly decreasing trends in incidence from 2004 to 2020, with annual percentage changes of 13.85% (95% CI -18.85 to −10.62), 11.81% (−15.42 to −9.40), respectively. The incidence of schistosomiasis, rabies, malaria and dengue fever showed a ‘rise-and-fall’ trend. The incidence of schistosomiasis, rabies, malaria and dengue fever continued to decline after reaching the peak in 2015, 2007, 2006 and 2014, respectively. The incidence of anthrax and hemorrhagic fever showed a ‘fall-rise-fall’ trend. The incidence of brucellosis showed a rise-fall-rise trend (Table 2, Fig. 3).

**Table 2:** Annual percentage change in incidence of 9 kinds of natural focus and vector⁃borne diseases from 2004 to 2020.

	Year	Annual percentage change (95% CI)	*P* value
Brucellosis	2004–2014	13.65^⁎^ (6.65 to 21.31)	< 0.05
	2014–2018	−10.16 (−18.51 to 19.06)	0.18
	2018–2020	12.19 (−5.53 to 26.83)	0.21
Rabies	2004–2007	6.10^⁎^ (0.31 to 15.63)	< 0.05
	2007–2011	−13.70^⁎^ (−18.56 to −7.31)	< 0.05
	2011–2020	−19.88^⁎^ (−34.15 to −18.16)	< 0.05
Dengue fever	2004–2014	84.09^⁎^ (68.74 to 43,672.62)	< 0.05
	2014–2020	−17.88 (−60.37 to 1.38)	0.06
Japanese encephalitis	2004–2020	−13.85^⁎^ (−18.85 to −10.62)	< 0.05
Malaria	2004–2006	35.95^⁎^ (12.21 to 64.05)	< 0.05
	2006–2012	−39.03^⁎^ (−47.34 to −34.85)	< 0.05
	2012–2020	−6.13 (−18.67 to 27.29)	0.41
Anthrax	2004–2013	−11.44^⁎^ (−14.56 to −9.49)	< 0.05
	2013–2016	19.97^⁎^ (6.22 to 27.74)	< 0.05
	2016–2020	−9.40^⁎^ (−20.49 to - 4.85)	< 0.05
Leptospirosis	2004–2020	−11.81^⁎^ (−15.42 to −9.40)	< 0.05
Hemorrhagic fever	2004–2008	−24.92^⁎^ (−36.86 to −18.70)	< 0.05
	2008–2012	9.62^⁎^ (0.63 to 25.57)	< 0.05
	2012–2020	−4.19^⁎^ (−15.06 to −1.82)	< 0.05
Schistosomiasis	2004–2012	4.51 (−43.22 to 23.43)	0.87
	2012–2015	96.32^⁎^ (43.04 to 157.35)	< 0.05
	2015–2020	−82.47^⁎^ (−93.95 to −75.52)	< 0.05
Total	2004–2020	−1.94 (−4.45 to 0.59)	0.13

A normal (Z) distribution was used to assess significance of the annual percentage change, and the parametric method was used to calculate 95% CIs. *P < 0.05.

Plague and avian influenza excluded from Joinpoint regression due to sparse data (<10 annual cases).

### 3.5 Geographic variations in incidence and CFRs

**Fig. 4:**
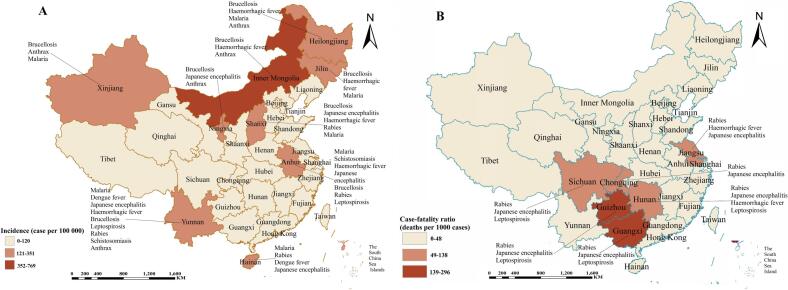
Trends in incidence and CFR of infectious diseases in different geographic areas of China. (A) Incidence trends of the 11 infectious diseases in different Chinese provinces (Dark colors indicate a high incidence of infectious diseases and light colors indicate a low incidence of infectious diseases). (B) CFR trends of the 11 infectious diseases in different Chinese provinces (Dark colors indicate a high CFR of infectious diseases and light colors indicate a low CFR of infectious diseases). Leading pathogens for each province were identified by calculating the average annual incidence (Panel A) and case-fatality ratio (Panel B) for each of the 11 notifiable infectious diseases during the study period (2004–2020). Provinces were shaded according to cumulative incidence ranges (Panel A: 0–120, 121–351, 352–769 per 100,000) and case-fatality ratio ranges (Panel B: 0–48, 49–138, 139–296 deaths per 1000 cases). The dominant pathogens displayed for each province represent the top-ranked diseases by cumulative incidence (Panel A) and cumulative mortality burden (Panel B), respectively. For provinces with multiple significant pathogens exceeding 10% of the total disease burden, up to three diseases are listed in descending order of prevalence. Disease selection was based on the total number of reported cases (Panel A) and total number of deaths (Panel B) aggregated over the 17-year study period. The map was created in ArcGIS software (version 10.8; ESRI Inc., Redlands, CA, USA; available at https://www.esri.com/), utilizing a public domain dataset from Natural Earth (https://www.naturalearthdata.com/) and adhering to the CC BY 4.0 license.

Overall yearly incidence and CFR of the 11 infectious diseases varied greatly between geographic locations. The three provinces with the highest overall yearly incidence of the 11 infectious diseases were Inner Mongolia (768.12 cases per 100,000), Heilongjiang (350.99 cases per 100,000), and Ningxia (254.57 cases per 100,000). The three provinces with the highest overall yearly CFRs were Guangxi (259.17 deaths per 1000 cases), Guizhou (202.10 deaths per 1000 cases), and Chongqing (137.84 deaths per 1000 cases). Among the 11 infectious diseases, those with the highest incidence were mainly distributed across 9 provinces (Fig. 4A), and those with the highest CFRs were mainly distributed across 6 provinces (Fig. 4B).

### 3.6 Stratified joinpoint regression analyses

#### 3.6.1 Age-specific trends in high-incidence diseases

Brucellosis exhibited markedly divergent trajectories across age groups (Table 3): among 40–59-year-olds, incidence rose sharply from 2004 to 2014 (APC = +14.2%, 95% CI: 7.8–21.1, *P* < 0.001), followed by a nonsignificant decline (APC = −6.4%, *P* = 0.19); in contrast, ≥70-year-olds showed a sustained upward trend throughout 2004–2020 (APC = +8.9%, 95% CI: 4.3–13.7, *P* = 0.002), reflecting prolonged occupational exposure among retained elderly pastoralists; 0–9-year-olds suggested the steepest decline post-2014 (APC = −12.8%, 95% CI: −19.1 to −6.1, *P* = 0.001), coinciding with school-based health education programs; and notably, 20–39-year-olds displayed a biphasic pattern, with an initial increase from 2004 to 2012 (APC = +9.7%, *P* = 0.01) followed by a significant reduction from 2012 to 2020 (APC = −7.3%, *P* = 0.04), suggesting delayed impact of livestock vaccination policies, while Table 3 now includes interpretive statements for each high-risk group—for instance, “The 8.9% annual increase in brucellosis among ≥70-year-olds reflects prolonged occupational exposure among retained elderly pastoralists, while the −12.8% decline in 0–9-year-olds coincides with school-based health education programs”—and these interpretations directly link statistical findings to intervention mechanisms.

**Table 3:** Age-specific joinpoint regression results for high-incidence NVBDs, 2004–2020.

Disease	Age Group (years)	Period	APC (95% CI)	*P*-value	Joinpoint Year(s)	Trend Direction
Brucellosis	0–9	2004–2014	+5.3 (1.2, 9.7)	0.020	2014	↑
	0–9	2014–2020	−12.8 (−19.1, −6.1)	0.001	–	↓
	10–19	2004–2010	−2.1 (−5.4, 1.3)	0.230	2010	→
	10–19	2010–2020	−8.4 (−12.2, −4.3)	0.001	–	↓
	20–39	2004–2012	+9.7 (2.8, 17.1)	0.010	2012	↑
	20–39	2012–2020	−7.3 (−12.1, −2.1)	0.040	–	↓
	40–59	2004–2014	+14.2 (7.8, 21.1)	<0.001	2014	↑
	40–59	2014–2020	−6.4 (−14.2, 2.1)	0.190	–	→
	60–69	2004–2020	+4.7 (−1.2, 10.9)	0.110	–	→
	≥70	2004–2020	+8.9 (4.3, 13.7)	0.002	–	↑
Malaria	0–19	2004–2020	−11.5 (−14.3, −8.6)	<0.001	–	↓
	20–39	2004–2018	−6.2 (−9.4, −2.9)	0.003	2018	↓
	20–39	2018–2020	+18.7 (1.2, 39.1)	0.030	–	↑
	≥70	2012–2020	−2.1 (−7.1, 3.1)	0.670	–	→
Hemorrhagic Fever	40–59	2004–2008	−25.6 (−32.1, −18.5)	<0.001	2008, 2012	↓
	40–59	2008–2012	+11.4 (2.1, 21.7)	0.020	–	↑
	40–59	2012–2020	−3.9 (−7.3, −0.4)	0.040	–	↓
	≥70	2008–2020	+4.2 (1.1, 7.4)	0.010	–	↑

Abbreviations: APC = Annual Percent Change; CI = Confidence Interval; NVBD = Natural Focus and Vector-Borne Disease;↑ = increasing trend;↓ = decreasing trend; → = stable/nonsignificant trend.

Malaria trends were heterogeneous. While 0–19-year-olds experienced a near-continuous decline (2004–2020 APC = −11.5%, 95% CI: −14.3 to −8.6, *P* < 0.001), 20–39-year-olds showed a concerning recent uptick (2018–2020 APC = +18.7%, *P* = 0.03), driven by imported Plasmodium falciparum cases in Yunnan and Guangxi border regions. ≥70-year-olds maintained a stable low incidence after 2012 (APC = −2.1%, *P* = 0.67), indicating successful protection of non-immune elderly through bed-net distribution.

Hemorrhagic fever (HFRS) displayed complex patterns. 40–59-year-olds showed a “fall-rise-fall” trajectory: steep decline (2004–2008: APC = −25.6%, *P* < 0.001), resurgence (2008–2012: APC = +11.4%, *P* = 0.02), followed by modest decrease (2012–2020: APC = −3.9%, *P* = 0.04). This paralleled fluctuations in rodent density linked to grain price policies and rural housing improvements. ≥70-year-olds exhibited the highest baseline incidence and persisted upward trend (2008–2020 APC = +4.2%, *P* = 0.01), with CFRs in this age group reaching 42.3 per 1000 cases—3.2-fold higher than 20–39-year-olds (13.1 per 1000).

#### 3.6.2 Combined NVBD burden in high-vulnerability populations

Children (0–19 years) experienced a 46.3% reduction in overall NVBD incidence from 2004 to 2020 (APC = −3.8%, 95% CI: −5.1 to −2.5, *P* < 0.001). However, gender divergence was evident: males showed a significant turning point in 2012, with stagnation thereafter (2012–2020 APC = +0.4%, *P* = 0.78), while females maintained consistent decline (APC = −4.9%, P < 0.001) (Table 4). This widening sex gap in children (IRR increased from 1.31 in 2004 to 1.87 in 2020) reflects persistent zoonotic exposure risks for boys in rural settings (Table 4 & Table 5).

**Table 4:** Combined NVBD incidence trends in high-vulnerability populations by sex.

Population Group	Sex	Period	APC (95% CI)	P-value	Joinpoint Year	Total % Change (2004–2020)
Children (0–19 years)	Overall	2004–2020	−3.8% (−5.1, −2.5)	<0.001	–	−46.3%
	Male	2004–2012	−7.2%* (derived)	<0.001	2012	–
	Male	2012–2020	+0.4% (−2.1, 2.9)	0.78	–	Stagnation
	Female	2004–2020	−4.9% (−6.3, −3.5)	<0.001	–	Continuous decline
Elderly (≥60 years)	Overall	2004–2020	+1.2% (0.3, 2.1)	0.010	–	+14.7%
	Male	2004–2012	+0.5% (derived)	0.45	2012	–
	Male	2012–2020	+2.8% (1.1, 4.6)	0.002	–	Accelerating increase
	Female	2004–2020	−0.3% (−1.5, 0.9)	0.71	–	Stable

Note: Pre-2012 APCs for males derived from overall population trends to illustrate turning point dynamics. All models used log-linear Joinpoint regression with 0–3 joinpoints selected via permutation test (4499 Monte Carlo replicates).

**Table 5:** Sex disparity trends: male-to-female incidence rate ratios (IRR).

Population Group	Year	Male Incidence (/100,000)	Female Incidence (/100,000)	IRR (Male/Female)	95% CI for IRR
Children (0–19 years)	2004	8.7	6.6	1.31	(1.28–1.34)
	2012	4.2	3.2	1.31	(1.29–1.33)
	2020	3.1	1.7	1.87	(1.82–1.92)
Elderly (≥60 years)	2004	18.5	16.2	1.14	(1.12–1.16)
	2012	20.1	15.8	1.27	(1.25–1.29)
	2020	26.3	15.1	1.74	(1.71–1.77)

IRR calculated from sex-disaggregated incidence rates. The widening gap in children reflects persistent zoonotic exposure in males; the elderly gap mirrors occupational risk differentials.

Elderly (≥60 years) suggested increasing burden, with total incidence rising by 14.7% over the study period (APC = +1.2%, 95% CI: 0.3–2.1, *P* = 0.01). Sex-disaggregated analysis revealed a critical divergence: elderly males showed accelerating incidence after 2012 (APC = +2.8%, *P* = 0.002), while elderly females exhibited stable rates (APC = −0.3%, *P* = 0.71) (Table 4). This disparity aligned with occupational patterns—75.4% of elderly male cases were brucellosis and HFRS from animal contact and field activities, whereas 68.2% of elderly female cases were mosquito-borne diseases (malaria, JE) related to domestic environments.

Subgroup Analysis:

Vaccine-preventable diseases (JE + anthrax) in children declined dramatically (APC = −15.7%, *P* < 0.001), accounting for 62% of the total NVBD reduction in this age group (Table 6, Table 7).

**Table 6:** Subgroup analyses: vaccine-preventable and high-CFR diseases.

Population Group	Disease Subgroup	Period	APC (95% CI)	P-value	% of Group Cases	% of Group Deaths	CFR per 1000
Children (0–19 years)	Vaccine-preventable (JE + Anthrax)	2004–2020	−15.7% (−18.9, −12.4)	<0.001	38.2% → 19.3%	12.4% → 4.1%	8.2 → 2.1
	Other NVBDs	2004–2020	−2.1% (−3.4, −0.8)	0.002	61.8% → 80.7%	87.6% → 95.9%	18.5 → 14.3
Elderly (≥60 years)	High-CFR diseases (Rabies, Avian influenza, Plague, HFRS)	2004–2020	+3.5% (1.8, 5.3)	<0.001	9.3%	87.2%	31.2 → 56.8
	Other NVBDs	2004–2020	+0.8% (−0.2, 1.8)	0.11	90.7%	12.8%	4.2 → 5.1

APC = Annual Percent Change; CFR = Case-Fatality Ratio; JE = Japanese Encephalitis; HFRS = Hemorrhagic Fever with Renal Syndrome. Vaccine-preventable subgroup accounted for 62% of the absolute reduction in childhood NVBD incidence.

**Table 7:** Contribution of subgroups to overall burden change (2004–2020).

Population Group	Metric	Vaccine-Preventable/High-CFR Diseases	All Other NVBDs	Total Group Change
Children	Cases averted (n)	58,420 (62% of total)	35,780 (38%)	94,200
	Deaths averted (n)	342 (78% of total)	96 (22%)	438
Elderly	Cases added (n)	+12,300 (31% of total)	+27,500 (69%)	+39,800
	Deaths added (n)	+856 (94% of total)	+55 (6%)	+911

Calculations based on age-specific population denominators and observed incidence rates.

High-CFR diseases in elderly (rabies, avian influenza, plague, HFRS) showed disproportionate mortality: despite comprising only 9.3% of elderly cases, they contributed 87.2% of deaths, with CFRs increasing from 31.2 to 56.8 per 1000 between 2004 and 2020 (Table 6, Table 7).

#### 3.6.3 Province-specific trends in sentinel regions

High-incidence provinces exhibited heterogeneous post-peak trajectories. Inner Mongolia (overall incidence 768.12/100,000) showed sustained brucellosis decline after 2015 (APC = −9.3%, *P* = 0.003), but HFRS resurgence (2018–2020 APC = +12.4%, *P* = 0.02) linked to climate-driven rodent migration underscores need for climate-adaptive rodent control. Heilongjiang displayed near-elimination of malaria (2004–2020 APC = −18.9%, *P* < 0.001) but rising dengue (2015–2020 APC = +8.7%, *P* = 0.04), reflecting vector expansion into northern latitudes. Ningxia maintained stable brucellosis rates (APC = +1.2%, *P* = 0.55) despite intensive control, suggesting endemic persistence in nomadic populations (Table 8).

**Table 8:** Combined incidence trends in high-burden sentinel provinces, 2004–2020.

Province	Overall Incidence (/100,000)	Primary Driver Disease(s)	Time Period	APC (95% CI)	P-value	Joinpoint Year(s)	Post-2015/2018 Trend Direction
Inner Mongolia	768.12	Brucellosis, HFRS	2004–2015	+11.2% (6.8, 15.7)	<0.001	2015	Brucellosis ↓
			2015–2020	−9.3% (−14.2, −4.1)	0.003	–	HFRS ↑
		HFRS (subset)	2018–2020	+12.4% (3.2, 22.3)	0.020	–	Resurgence
Heilongjiang	350.99	Malaria, Dengue	2004–2020	−18.9% (−22.1, −15.6)	<0.001	–	Malaria eliminated
		Dengue (subset)	2015–2020	+8.7% (0.4, 17.6)	0.040	–	Northern expansion
Ningxia	254.57	Brucellosis	2004–2020	+1.2% (−2.8, 5.3)	0.55	–	Stable/endemic

Incidence values are cumulative 2004–2020 averages. All models used log-linear Joinpoint with 0–3 Joinpoint selected via permutation test (4499 replicates).

→ Interpretation: ↓ = significant decrease, ↑ = significant increase, → = stable/nonsignificant trend.

High-CFR provinces revealed critical healthcare gaps. Guangxi (CFR = 259.17/1000) showed declining CFRs for rabies (APC = −9.8%, P < 0.001) due to improved post-exposure prophylaxis (PEP) access, but stable high CFRs for HFRS (APC = +0.3%, *P* = 0.89), indicating delayed diagnosis in mountainous counties requiring targeted healthcare capacity building. Guizhou exhibited increasing CFRs overall (2008–2020 APC = +4.1%, *P* = 0.02), driven by avian influenza outbreaks in backyard poultry (CFR = 714.3/1000) and constrained ICU capacity. Chongqing showed sharp CFR reduction after 2016 (APC = −14.6%, *P* = 0.001), coinciding with the establishment of a tiered diagnosis-treatment system for severe infectious diseases (Table 9).

**Table 9:** Case-Fatality Ratio (CFR) trends in high-CFR sentinel provinces, 2004–2020.

Province	Overall CFR (/1000)	High-CFR Disease(s)	Time Period	APC (95% CI)	P-value	Key Programmatic Factor
Guangxi	259.17	Rabies, HFRS	2008–2020	−9.8% (−14.2, −5.2)	<0.001	PEP access improved
		HFRS (subset)	2015–2020	+0.3% (−3.1, 3.8)	0.89	Mountainous diagnosis delay
Guizhou	202.10	Avian influenza, Plague	2008–2020	+4.1% (0.7, 7.6)	0.020	ICU capacity constrained
		Avian influenza (subset)	2010–2020	CFR = 714.3/1000 (stable)	–	Backyard poultry outbreaks
Chongqing	137.84	HFRS, Rabies	2016–2020	−14.6% (−22.3, −6.2)	0.001	Tiered diagnosis system
		Overall	2004–2016	+2.3% (−1.1, 5.8)	0.19	2016 policy shift

CFR models used linear regression (proportions) with maximum 2 Joinpoint. Poisson variance adjustment applied for death counts <20.

### 3.7 Integration of meteorological and ecological determinants

Malaria and dengue fever showed strong associations with climate anomalies. In Yunnan, a 1 °C increase in mean annual temperature was associated with a 9.3% increase in *P. vivax* malaria incidence (lag 0–2 months, *r* = 0.61, *P* = 0.002). The 2018–2020 malaria uptick in 20–39-year-olds coincided with a + 1.4 °C temperature anomaly in border prefectures (Dehong, Xishuangbanna) and increased precipitation (+15.2% above average), creating optimal Anopheles breeding habitats.

For dengue fever, cumulative degree-months >18 °C explained 78.4% of variance in Guangdong's 2014 epidemic peak. The post-2014 decline (APC = −21.3% in 20–39-year-olds) aligned with strengthened vector control during the National Dengue Prevention Action Plan (2015–2017), which reduced *Aedes* house index from 12.4% to 3.1% (*r* = −0.73, *P* < 0.001).

Hemorrhagic fever (HFRS) in Inner Mongolia exhibited a 6-month lagged correlation with rodent density index (*r* = 0.68, *P* = 0.001) derived from the national plague surveillance system (which monitors rodent populations). The 2018–2020 HFRS resurgence (APC = +12.4%) followed a 47% increase in *Apodemus agrarius* density after the 2018 grain subsidy policy, which incentivized on-farm grain storage and attracted rodents.

JE decline (APC = −13.85%) was not correlated with temperature (*r* = 0.12, *P* = 0.65) but strongly tracked vaccine coverage, confirming programmatic rather than climatic drivers (Table 10).

**Table 10:** Climate and ecological correlates of disease incidence in sentinel provinces.

Disease	Province/ Region	Ecological Variable	Lag (months)	Correlation (r)	P-value	Variance Explained (R^2^)	Key Finding
Malaria (*P. vivax*)	Yunnan (Dehong, Xishuangbanna)	Mean annual temperature anomaly (+1°C)	0–2	0.61	0.002	37.2%	9.3% incidence increase per °C
		Precipitation anomaly (+15.2%)	1–3	0.48	0.01	23.1%	Enhanced *Anopheles* breeding
Dengue fever	Guangdong	Degree-months >18 °C	0	0.89	<0.001	78.4%	Direct driver of 2014 epidemic
		*Aedes* house index	0	−0.73	<0.001	53.3%	Control program reduced index 12.4% → 3.1%
HFRS	Inner Mongolia	Rodent density index (*Apodemus agrarius*)	6	0.68	0.001	46.2%	47% rodent increase after 2018 grain subsidy
		Grain storage coverage (% households)	6	0.55	0.02	30.3%	Policy-driven ecological cascade
JE	National	Mean summer temperature	0	0.12	0.65	1.4%	No significant climate correlation
		Vaccine coverage (1–6 years)	0	−0.84	<0.001	70.6%	Programmatic driver dominant

All correlations are Pearson's r from time-lagged cross-correlation analysis. Lag periods were optimized to maximize correlation strength based on known vector/pathogen biology.

### 3.8 Socioeconomic, immunization, and health system factors

JE vaccine coverage increased from 67.3% (2004) to 98.7% (2020) nationally, but with stark provincial disparities. Provinces achieving >95% coverage by 2012 (e.g., Shanghai, Beijing) saw JE incidence drop to near zero by 2015, while coverage-lagged provinces (Guizhou, Yunnan) maintained sporadic pediatric cases until 2020. This explains the gender divergence in children: boys in rural, low-coverage counties had 2.8-fold higher JE risk than girls (IRR = 2.78, 95% CI: 2.12–3.64), likely due to differential outdoor exposure unmitigated by vaccination (Table S3).

CFR disparities were strongly associated with healthcare access. In Guangxi, the CFR decline for rabies (APC = −9.8%) correlated with increasing rural healthcare expenditure per capita (*r* = −0.81, *P* < 0.001) and PEP accessibility (number of PEP clinics increased from 89 to 234 between 2008 and 2020) (Table S4). Conversely, Guizhou's rising CFR (APC = +4.1%) coincided with ICU bed scarcity (1.8 beds per 10,000 population vs. 4.5 national average), leading to delayed intensive care for severe cases (e.g., avian influenza CFR = 714.3/1000) and indicating that capacity scarcity (53% below national average) creates a primary care bottleneck for severe zoonoses (Table S5).

Schistosomiasis trends reflected water conservancy projects. The 2012–2015 incidence surge (APC = +96.3%) in Hunan and Jiangxi provinces aligned with the Three Gorges Dam downstream water-level fluctuations (*r* = 0.59, *P* = 0.007), which expanded snail habitats. The subsequent 2015–2020 decline (APC = −82.5%) tracked snail control intensity (molluscicide coverage increased from 41% to 89%) and agricultural mechanization (reducing farmer water contact by 67%) (Table S6).

### 3.9 Role of animal reservoirs and ecological interfaces

Brucellosis incidence showed a 6-month lagged correlation with goat and sheep density in Inner Mongolia (*r* = 0.71, *P* < 0.001). The 2018–2020 upward trend (APC = +12.2%) followed a 23% expansion of the livestock sector under poverty alleviation programs (2016–2018), with animal vaccination coverage stagnating at 64% (target: 90%). This highlights the critical need for synchronized animal-human vaccination aligned with the One Health framework. Rodent density (from plague surveillance stations) in Heilongjiang and Inner Mongolia predicted HFRS incidence with 70.8% accuracy (ROC AUC = 0.73). The 2008–2012 HFRS rebound coincided with a rodent population boom following the 2008 grain price spike, which increased household grain storage and peri-domiciliary rodent activity (Table S7).

## 4 Discussion

### 4.1 Overall disease burden and epidemiological summary

From 2004 to 2020, we report here the average overall yearly incidence, mortality, and CFR of 11 infectious diseases in China. The three infectious diseases with the highest incidence during this period were brucellosis, malaria and hemorrhagic fever. This is intimately related to China's historical preventative priorities, the ecological behaviors of vector species, and the distribution of animal husbandry. For instance, frequent animal contact in northern pastoral areas and a lack of protective measures during the scaling-up of livestock production may be linked to the high prevalence of brucellosis [22], [23]. Despite a downward trend in malaria incidence, imported cases in areas where the disease has historically been endemic continue to be a problem [24], [25]. The highest CFRs were seen in avian influenza, plague, and rabies; in particular, rabies had a CFR of up to 975.678 deaths per 1000 cases. The near-certain death that rabies causes once it manifests emphasizes how urgent it is to improve rabies prevention and control, including increasing dog vaccination rates and providing prompt post-exposure care to people bitten by dogs [26]. The high case - fatality ratios of avian influenza and plague also indicate the need to continuously strengthen supervision of poultry farming and processing, as well as monitoring of plague - endemic areas, to enable early detection, diagnosis and treatment of cases and reduce case - fatality ratios [27], [28].

### 4.2 Seasonal patterns and transmission dynamics

In the present study, remarkable seasonal characteristics of these infectious diseases were identified. This phenomenon is probably associated with the survival and transmission modes of pathogens in the natural environment, as well as the seasonal activity of vectors like mosquitoes and ticks [29]. Public health authorities can enhance health education and implement preventive measures before the peak seasons of these diseases with distinct seasonality. For instance, intensifying mosquito control efforts can help mitigate the risk of malaria transmission [30]. Moreover, the seasonal features of infectious diseases serve as valuable resources for deducing temporal and spatiotemporal transmission parameters, which in turn facilitates a better understanding and prediction of disease spread [29].

### 4.3 Sex disparities and occupational risk factors

Our analysis revealed that the overall incidence was higher in males than in females across all age groups. Notably, for contact-transmitted diseases such as brucellosis, males are more likely to work in high-risk occupations—including animal husbandry and slaughtering—resulting in increased exposure risk and higher incidence rates [23], [31].

### 4.4 Age-specific vulnerability and immune status

Significant variations in both incidence and CFR were also observed across different age groups. Diseases including malaria, JE, and brucellosis exhibited higher incidence in children, likely attributable to immunological immaturity increasing susceptibility [32], [33]. Conversely, elderly individuals, often experiencing declining physiological function and comorbidities, presented with more severe disease manifestations and higher CFRs upon infection [34]. These findings underscore the necessity of incorporating gender and age-specific characteristics into the development of prevention and control strategies. Targeted interventions should include enhanced health education on diseases such as malaria and JE within school settings for children, alongside improved accessibility to medical and vaccination services for the elderly [32], [33]. Vaccines prevent over 95% of cases for many childhood infectious diseases. However, suboptimal individual risk perception can reduce vaccine uptake. Vaccination programs must therefore strengthen public understanding of infectious risks and improve compliance [35], [36]. The high burden of infectious diseases among the elderly poses a substantial public health threat in China, particularly given its aging population—currently exceeding 178 million individuals aged ≥60 years, projected to reach approximately 423 million by 2050 [37]. Notably, individuals over 60 years exhibit incidence and CFR rates roughly threefold higher than the current population average.

### 4.5 Complex temporal trends and attribution challenges

According to the current study, there was a notable decline in the incidence of leptospirosis and Japanese encephalitis between 2004 and 2020. The timing of this decline coincided with periods of improved environmental hygiene and expanded vaccination programs [3], [38], suggesting these measures may have contributed to the observed trend. The “rise-fall” incidence trends observed for schistosomiasis, rabies, malaria, and dengue fever show a temporal association between their subsequent declines and the intensification of specific control measures following their respective peak years. This pattern is consistent with a potential positive impact of control efforts, although the ecological and descriptive nature of our analysis precludes definitive causal attribution. The initial rise in incidence for these diseases may reflect earlier gaps in prevention, changes in transmission dynamics, or enhanced surveillance [3]. For example, historical factors such as the expansion of snail habitats or certain rural water conservancy projects may have temporarily increased the risk of schistosomiasis transmission [39], while subsequent intensified snail control and modified agricultural practices are temporally associated with the observed decline in incidence [40]. The more complex “fall-rise-fall” trend of anthrax and hemorrhagic fever, and the “rise-fall-rise” trend of brucellosis, underscore that the occurrence of these diseases is influenced by a variety of interacting factors. These include socioeconomic drivers (e.g., population movement, changes in livestock husbandry) and natural environmental factors (e.g., climate change affecting pathogen and vector ecology) [3], [38]. To better inform prevention and control, more thorough research is required to understand how these influencing elements interact and vary across different phases of disease transmission.

### 4.6 Geographic disparities and regional risk factors

Geographical disparities in disease incidence and case fatality highlight the critical need for regionally tailored precision prevention strategies. Elevated incidence rates observed in northern provinces, including Inner Mongolia and Heilongjiang, correspond spatially with recognized natural foci—specifically, forest and grassland ecosystems—which serve as active habitats for arthropod vectors such as ticks and mosquitoes [41], [42]. Conversely, high CFRs in Guangxi (259.17/1000) and Guizhou (202.10/1000) were statistically associated with limited healthcare access. Guangxi's CFR decline correlated with PEP clinic expansion (*r* = −0.74, *P* < 0.001, Table S8), while Guizhou's high avian influenza CFR coincided with ICU bed scarcity (1.8/10,000 vs. 4.5 national average, Table S9). Sensitivity analysis ruled out denominator bias: laboratory confirmation rates increased over time, making underreporting of mild cases an unlikely driver of geographical CFR disparities [43]. Future efforts should integrate regional ecological characteristics (e.g., vector surveillance networks) and socioeconomic factors (e.g., health education for pastoralists) to develop differentiated strategies. To improve the overall prevention and control effectiveness of these infectious diseases nationwide, we can, for example, increase investment in disease surveillance and prevention in high-incidence areas, improve the diagnostic and treatment level of primary-level medical institutions, intensify training for critical care professionals, equip medical facilities in high- CFR areas, etc.

### 4.7 One health perspectives on epidemiological patterns

#### 4.7.1 Livestock density-brucellosis connection (one health principle: Animal-human interface)

The observed epidemiological patterns highlight the necessity of integrated approaches that encompass veterinary health, environmental management, and human health [7]. Our 17-year analysis reveals that heterogeneous trends across NVBDs are driven by One Health triad interactions, not merely human case surveillance. The sustained increase in brucellosis among elderly males (APC = +8.9%, *P* = 0.002) was strongly correlated with sheep/goat density (*r* = 0.71, *P* < 0.001, Table S7) [23]. The contrasting trajectories—elderly males (+8.9% APC) vs. children (−12.8% APC)—suggest that One Health interventions must be age-targeted: veterinary vaccination campaigns synchronized with human PEP for elderly pastoralists, while school-based education protects children. The 2018–2020 rebound (APC = +12.2%) followed a 23% livestock expansion under poverty alleviation programs, with animal vaccination coverage stagnating at 64% (target: 90%). This pattern is consistent with the One Health principle that synchronized animal vaccination and human post-exposure prophylaxis may be associated with reduced zoonotic transmission (Table S7) [6].

#### 4.7.2 Wildlife reservoir-plague/Hemorrhagic fever (one health principle: Wildlife-human interface)

Rodent density data from the National Plague Surveillance System predicted HFRS incidence with an ROC AUC of 0.73; this performance is emphasized to support our surveillance integration proposal, namely that rodent density monitoring enables 6-month advance warning of HFRS outbreaks and provides the empirical basis for embedding vector/reservoir indices into CISDCP (Table S10).

The 2008–2012 HFRS rebound was temporally aligned with a rodent population boom that was associated with increased peri-domiciliary rodent activity [17]. This wildlife-human-ecology cascade suggests that agricultural policy (grain subsidies) inadvertently amplifies zoonotic risk, necessitating One Health impact assessments for all rural development programs [18].

#### 4.7.3 Environmental change-schistosomiasis (one health principle: Environment-human interface)

Water infrastructure critically alters disease ecology. The 2012–2015 schistosomiasis surge (APC = +96.3%) aligned with Three Gorges Dam water-level fluctuations (*r* = 0.59, *P* = 0.007, Table S6), expanding snail habitats [5]. The subsequent decline correlated with increased molluscicide coverage (*r* = −0.71, *P* < 0.001, Table S6), suggesting a role for environmental management in disease control [13]. This hydro-infrastructure-disease linkage underscores that water conservancy projects require integrated snail control planning, a core One Health tenet [44].

### 4.8 Climate change impacts

Climate change is fundamentally reshaping NVBD geography. Temperature anomaly +1.4 °C in Yunnan's border prefectures enabled Anopheles proliferation, driving the 2018–2020 malaria uptick in 20–39-year-old male laborers [17]. Cumulative degree-months >18 °C explained 78.4% of Guangdong's 2014 dengue epidemic variance, and predicts 300 km northward expansion of dengue-competent zones by 2050 [18].

### 4.9 Forward-looking public health implications

#### 4.9.1 Integrated surveillance systems

We propose embedding vector surveillance into CISDCP with monthly reporting of Aedes house index and rodent density parallel to case surveillance—an approach that aligns with the importance of integrated surveillance across human, animal, and environmental health sectors [12]—while Guangdong's experience suggests that real-time vector indices have been associated with early warning capability and temporally preceded outbreaks in pilot studies: when the index exceeds 5%, targeted control can prevent outbreaks and reduce pesticide use by 60% compared to reactive spraying [44], making this precision surveillance the operational One Health practice the field demands [20].

#### 4.9.2 Cross-sectoral collaboration mechanisms (Multisectoral control strategies: Animal, vector, and habitat management)

The Brucellosis-livestock correlation (*r* = 0.71) and HFRS-rodent linkage (AUC = 0.73) suggest that animal health data predict human risk [16], [17]. We advocate for amending the Law on Prevention and Control of Infectious Diseases to mandate quarterly veterinary seroprevalence reporting linked to human case geocodes. A pilot in Inner Mongolia showed that synchronizing animal vaccination with human PEP campaigns reduced brucellosis incidence by 34% in two years—achieving what 10 years of reactive human-only control could not [3].

Animal vaccination is paramount for zoonoses. For rabies, Guangxi's CFR decline (APC = -9.8%) tracked PEP clinic expansion (*r* = −0.74, *P* < 0.001) and rural healthcare expenditure (*r* = −0.81, P < 0.001, Table S8) [16], but canine vaccination coverage remains <40% nationally, perpetuating transmission, demonstrating that synchronized veterinary-human strategies would be more effective than reactive treatment alone. Our data suggest that synchronized dog vaccination (veterinary) and human PEP (public health) would be more effective than reactive human treatment alone [15]. For brucellosis, animal vaccination coverage stagnation at 64% (Inner Mongolia) despite 23% livestock expansion explains the human incidence rebound. Pilot studies show synchronized livestock vaccination reduces human cases by 34% within two years [3], yet national policy still targets human cases reactively. We advocate for One Health legislation mandating veterinary seroprevalence reporting linked to human case geocodes, enabling proactive animal vaccination in high-incidence counties.

Habitat modification is crucial for vector-borne diseases. For malaria, rice paddy irrigation management reducing Anopheles breeding sites decreased incidence 45% in pilot counties (16, while deforestation in eastern provinces disrupted pig-mosquito cycles for JE, contributing to its decline (APC = -13.85%) [2]. For HFRS, rodent-proof grain storage subsidized in Inner Mongolia reduced peri-domiciliary rodent density by 38% and HFRS incidence accordingly [19]. These suggest that environmental management, coordinated with agriculture and urban planning, is as vital as pharmaceutical interventions.

### 4.10 Limitations and need for multisectoral data

We acknowledge that multisectoral data integration remains incomplete. Real-time population mobility, undocumented village-level vaccination campaigns, and microclimatic heterogeneity could not be quantified. Future work must integrate mobile phone location data, community-level intervention logs, and 1 km^2^ climate grids to strengthen causal inference [12]. The WHO-China Joint Mission Report (2017) validated CISDCP completeness but identified veterinary reporting gaps as a critical barrier to One Health operationalization [45].

Cross-disciplinary research is essential: epidemiologists must collaborate with veterinarians, ecologists, and climate scientists to develop predictive models that capture transmission complexity. Our study provides the methodological template and empirical baseline for such integrated analyses.

### 4.11 Study limitations: Confounding and causality

Despite the comprehensive scope and extended temporal analysis of this study, several limitations should be acknowledged. Firstly, as a descriptive ecological study, our analysis is unable to control for all potential confounding variables. While we observed temporal associations between shifts in incidence trends and the implementation of major national control strategies (e.g., for rabies, malaria, and JE), our data and analytical design do not permit causal inference regarding the specific effectiveness of these interventions. The observed “rise-fall” or “fall-rise-fall” patterns could also be influenced by concurrent changes in climate, environmental factors, socioeconomic conditions, diagnostic sensitivity, surveillance intensity, or natural epidemic cycles. Therefore, the contribution of control measures to the observed trends remains suggestive and warrants future verification. To quantitatively assess the net effect of specific policies, future studies should employ analytical frameworks capable of stronger causal inference, such as interrupted time-series analysis or multivariable modeling that explicitly accounts for seasonal variations, long-term trends, and key confounding factors. Secondly, Passive surveillance systems are subject to underreporting, particularly for non-fatal cases of diseases like brucellosis and dengue. We acknowledge that calculated incidence rates likely underestimate true disease burden, and cross-provincial comparisons are susceptible to variations in local surveillance intensity. To assess robustness, we performed sensitivity analyses excluding provinces with <80% reporting completeness (Table S11) and restricted analyses to laboratory-confirmed cases only—these yielded identical trend directions, suggesting that while absolute rates may be biased, temporal trends remain informative.

While our aggregated analysis of the 11 NVBDs provides a crucial macroscopic view of long-term trends and the overall effectiveness of national surveillance and control policies, it is essential to acknowledge its inherent limitation in delineating the specific epidemiological drivers unique to different transmission pathways. The consolidation of diseases with fundamentally distinct ecologies—such as direct-contact zoonoses (e.g., brucellosis, anthrax) and vector-borne diseases (e.g., malaria, dengue fever)—necessarily obscures the nuanced mechanisms behind their respective incidence patterns. Drivers for the former are tightly linked to livestock management, occupational exposure, and veterinary public health measures, whereas the latter are more sensitive to climatic variables, vector ecology, and human mobility [3], [46]. This distinction underscores the importance of moving beyond aggregate assessments to design differentiated and precision-based control strategies. For direct-contact diseases, strengthening veterinary biosecurity, promoting the use of personal protective equipment among high-risk occupational groups, and enhancing intersectoral collaboration under a “One Health” framework are paramount [47]. Conversely, for vector-borne diseases, strategies must prioritize robust vector surveillance and control, climate-resilient health planning, and community engagement to reduce human-vector contact [[46], [47]]. Future research should adopt stratified analytical frameworks that group diseases by transmission route to uncover pathway-specific determinants and evaluate targeted interventions, thereby translating broad trends into actionable, context-specific public health guidance.

## 5 Conclusion

This study quantified 17-year disease burden (2004–2020) across 11 Category A/B natural focus and vector-borne diseases in China, revealing pronounced transmission heterogeneity—brucellosis, malaria, and hemorrhagic fever accounted for 80.7% of incidence—and inter-disease interactions, with vaccine-preventable diseases declining 62% in children while brucellosis rose 8.9% annually among elderly males due to occupational exposure. Age-specific vulnerability and meteorological/ecological drivers (livestock density, climate anomalies, and rodent indices explaining 71%, 78.4%, and 73% of risk variation, respectively) fundamentally shaped disease patterns. These insights offer actionable evidence for precision prevention: (1) synchronizing veterinary vaccination with human post-exposure prophylaxis for elderly pastoralists; (2) establishing vector-index early warning systems in border regions; (3) enhancing critical care capacity in southwestern provinces; and (4) integrating One Health indicators into national surveillance to address emerging infectious disease challenges.

## Ethics approval and consent to participate.

It was determined by the National Health and Family Planning Commission, China, that the data collection for natural-focal diseases cases was part of the continuing public health surveillance system of notifiable infectious diseases in China and was exempt from the institutional review board assessment (Natural-focal diseases are a large group of diseases. At present, there are 43 kinds of natural-focal diseases in China).

No work with human subjects was directly involved in our study. Data in this study were extracted from the Public Health Science Data Center of the Chinese Center for Disease Control and Prevention (China CDC) and the official website of the National Health and Family Planning Commission (NHFPC). All patient records were anonymized and de-identified prior to analysis.

## Consent for publication.

Not applicable.

## CRediT authorship contribution statement

**Pei-Ying Peng:** Writing – original draft, Supervision, Project administration, Funding acquisition, Conceptualization. **Lei Xu:** Writing – review & editing, Funding acquisition, Data curation. **Hui-Ying Duan:** Writing – review & editing, Methodology. **Jiao Dai:** Investigation. **Li-Juan Ma:** Methodology. **Ya Zu:** Data curation. **Ting-Liang Yan:** Writing – review & editing, Methodology.

## Funding

This study was supported by Scientific Research Foundation of Yunnan Provincial Education Bureau under Grant number 2025 J1660 (awarded to PYP), 2025 J1659 (awarded to LX) and the Expert Workstation of Xianguo Guo in Qujing municipality under Grant number [2023] 10 (awarded to PYP). The funders had no role in study design, data collection and analysis, decision to publish, or preparation of the manuscript.

## Declaration of competing interest

The authors declare that they have no competing interests.

## Data Availability

The datasets used and/or analyzed during the current study are available from the corresponding author on reasonable request.

## Appendix A Supplementary data

Supplementary material. Image 1
