# Supplementary material for: One health perspectives on the epidemiological features and changing incidence of natural focus and vector-borne infectious diseases in China: An observational trend study
Source: One Health. 2026 Feb 21;22:101373. doi: 10.1016/j.onehlt.2026.101373 (PMC13080507; doi:10.1016/j.onehlt.2026.101373)
Supplement: Supplementary file 1 — Supplementary material. [file mmc1.docx]

**Table S1**

Multivariable Joinpoint models adjusting for surveillance quality indicators

| Disease | Model Type | Period | APC (Unadjusted) | APC (Adjusted for Reporting Completeness) | APC (Adjusted for Lab Confirmation Rate) | Change in APC (%) | P-value (Adjusted) |
| --- | --- | --- | --- | --- | --- | --- | --- |
| Brucellosis | Log-Linear | 2004–2014 | +14.2% (7.8, 21.1) | +13.8% (7.2, 20.6) | +12.9% (6.5, 19.8) | −3% to −9% | <0.001 |
|  |  | 2014–2020 | −6.4% (−14.2, 2.1) | −6.1% (−13.8, 2.5) | −5.7% (−13.1, 3.1) | −5% to −11% | 0.19 |
| Malaria | Log-Linear | 2004–2020 | −11.5% (−14.3, −8.6) | −10.9% (−13.8, −7.9) | −10.2% (−13.1, −7.2) | −5% to −11% | <0.001 |
| HFRS | Log-Linear | 2004–2008 | −25.6% (−32.1, −18.5) | −24.1% (−30.8, −16.9) | −23.8% (−30.2, −16.7) | −6% to −7% | <0.001 |
|  |  | 2008–2012 | +11.4% (2.1, 21.7) | +10.8% (1.5, 20.9) | +10.2% (0.8, 20.5) | −5% to −11% | 0.03 |
| Dengue | Log-Linear | 2014–2020 | −21.3% (−28.7, −13.1) | −20.5% (−27.9, −12.3) | −19.8% (−27.3, −11.5) | −4% to −7% | <0.001 |
| Schistosomiasis | Log-Linear | 2012–2015 | +96.3% (43.0, 157.4) | +89.2% (38.1, 149.8) | +91.5% (41.2, 152.3) | −7% to −5% | <0.001 |
|  |  | 2015–2020 | −82.5% (−93.9, −75.5) | −79.8% (−92.1, −72.1) | −80.2% (−92.6, −73.4) | −3% to −3% | <0.001 |

Adjustment method: Reporting completeness weight = province's annual CDC audit score (0.8–1.0); Lab confirmation rate weight = % of cases with laboratory confirmation. Weights applied via inverse probability weighting in Joinpoint model.

**Table S2**

Sensitivity analysis using Poisson regression for rare diseases

| Disease | Period | Original Joinpoint APC (95% CI) | Poisson APC (95% CI) | % Change in APC | Interpretation |
| --- | --- | --- | --- | --- | --- |
| Plague | 2004–2020 | Not applicable | Not applicable | - | Plague was excluded due to sparse data |
| Avian Influenza | 2005–2020 | +2.3% (-0.1, 4.7) | +2.1% (-0.2, 4.4) | -8.7% | Slight increase, robust to zero values |
| Anthrax | 2004–2017 | +1.5% (-0.2, 3.2) | +1.6% (-0.1, 3.3) | +6.7% | Slight increase, robust to zero values |

"Not applicable" indicates that Poisson regression was not used due to the nature of the data (e.g., insufficient data points for stable estimation).

The % Change in APC column shows the difference between the APCs from the original Joinpoint analysis and the Poisson regression analysis.

Interpretation column provides a brief summary of the findings from the sensitivity analysis.

**Table S3**

Japanese Encephalitis vaccine coverage and incidence by province, 2012 vs. 2015

| Province | JE Vaccine coverage 2012 (%) | JE Incidence 2012 (/100000) | JE Vaccine coverage 2015 (%) | JE Incidence 2015 (/100000) | % Incidence reduction | Achievement status |
| --- | --- | --- | --- | --- | --- | --- |
| Shanghai | 98.2 | 0.02 | 99.8 | 0.00 | 100% | Early elimination |
| Beijing | 97.5 | 0.03 | 99.5 | 0.00 | 100% | Early elimination |
| Jiangsu | 96.8 | 0.08 | 98.9 | 0.01 | 87.5% | Near elimination |
| Guangdong | 94.3 | 0.15 | 97.2 | 0.03 | 80.0% | Near elimination |
| Sichuan | 82.1 | 0.34 | 91.5 | 0.12 | 64.7% | Delayed decline |
| Hunan | 76.4 | 0.45 | 89.3 | 0.18 | 60.0% | Delayed decline |
| Yunnan | 68.7 | 0.67 | 78.2 | 0.28 | 58.2% | Lag elimination |
| Guizhou | 61.2 | 0.89 | 74.5 | 0.41 | 53.9% | Lag elimination |
| National | 84.3 | 0.18 | 92.7 | 0.08 | 55.6% | - |

Coverage calculated as (doses administered / target population age 1–6 years) × 100. Incidence rates reflect confirmed cases per 100,000 population. Early elimination provinces achieved >95% coverage by 2012 and zero indigenous cases by 2015.

**Table S4**

Healthcare access indicators and rabies CFR in Guangxi

| Year | Rural Healthcare Expenditure per Capita (¥) | PEP Clinics (n) | Mean Distance to PEP Clinic (km) | Rabies Cases (n) | Rabies Deaths (n) | CFR (/1,000) | APC (2008–2020) |
| --- | --- | --- | --- | --- | --- | --- | --- |
| 2008 | 850 | 89 | 45 | 142 | 138 | 971.8 | - |
| 2010 | 1,120 | 124 | 38 | 118 | 113 | 957.6 | - |
| 2012 | 1,450 | 156 | 32 | 87 | 81 | 931.0 | - |
| 2014 | 1,780 | 189 | 26 | 65 | 58 | 892.3 | - |
| 2016 | 2,050 | 212 | 21 | 48 | 41 | 854.2 | - |
| 2018 | 2,240 | 228 | 19 | 34 | 27 | 794.1 | - |
| 2020 | 2,340 | 234 | 18 | 28 | 21 | 750.0 | -9.8%* |

PEP = Post-Exposure Prophylaxis. CFR = Case-Fatality Ratio. Correlation between healthcare expenditure and CFR: r = -0.81 (P < 0.001). APC calculated by Joinpoint regression with 0 joinpoints (monotonic decline). *P < 0.001.

**Table S5**

Healthcare capacity vs. Avian Influenza CFR in Guizhou, 2010–2020

| Indicator | 2010 | 2015 | 2020 | Correlation with CFR (r) | P-value |
| --- | --- | --- | --- | --- | --- |
| ICU beds per 10,000 population | 1.2 | 1.5 | 2.4 | -0.68 | 0.03 |
| Mechanical ventilators per 100,000 | 2.8 | 3.2 | 5.1 | -0.72 | 0.02 |
| Mean time to oseltamivir initiation (hours) | 48 | 36 | 24 | +0.59 | 0.05 |
| Avian Influenza cases (n) | 8 | 12 | 7 | - | - |
| Avian Influenza deaths (n) | 6 | 9 | 5 | - | - |
| CFR (/1,000) | 750.0 | 750.0 | 714.3 | - | - |

CFR remained persistently high despite modest capacity improvements, indicating bottleneck at primary care level (delayed diagnosis). National average ICU beds = 4.5/10,000; Guizhou remains 47% below.

**Table S6**

Schistosomiasis control inputs and outcomes in Hunan & Jiangxi, 2004–2020

| Year | Water level fluctuation (m)* | Snail habitat area (km²) | Molluscicide coverage (%) | Agricultural mechanization (%) | Farmer water contact (hrs/week) | Schistosomiasis incidence (/100000) |
| --- | --- | --- | --- | --- | --- | --- |
| 2004 | Baseline (145) | 1,245 | 38% | 28% | 18.5 | 4.2 |
| 2008 | +2.1 | 1,387 | 41% | 35% | 16.2 | 3.8 |
| 2012 | +5.3 | 1,568 | 45% | 48% | 14.8 | 4.5 |
| 2015 | +7.8 | 1,734 | 89% | 62% | 11.3 | 8.7** |
| 2018 | +6.2 | 892 | 92% | 78% | 6.1 | 2.1 |
| 2020 | +4.1 | 567 | 94% | 89% | 4.8 | 0.9 |

Data Sources:

Water level: Three Gorges Dam Authority, daily water level records (Wusong datum)

Snail habitat: National Schistosomiasis Reference Laboratory, annual snail survey (collateral sampling)

Molluscicide coverage: Provincial CDC procurement records (niclosamide distribution)

Agricultural mechanization: Ministry of Agriculture tractor registry data

Farmer water contact: Schistosomiasis sentinel surveillance household surveys (n=2,400 households/year)

Incidence: CISDCP confirmed cases (serology + stool microscopy)

*Water level relative to Three Gorges Dam baseline (145m). **2012–2015 surge (APC=+96.3%) coincided with peak water-level fluctuation.

Statistical Correlations:

Water fluctuation vs. snail habitat: r = 0.77 (P < 0.001)

Snail habitat vs. incidence: r = 0.59 (P = 0.007)

Mechanization vs. water contact: r = −0.84 (P < 0.001)

Molluscicide vs. incidence (lag 1yr): r = −0.71 (P < 0.001)

Key insight: The 2012–2015 surge coincided with peak water fluctuation and snail expansion, while the 2015–2020 decline tracked intensified control measures, demonstrating the hydro-infrastructure-disease linkage that requires integrated One Health planning.

**Table S7**

Livestock density and Brucellosis correlation in pastoral Provinces (2004-2020)

| Province | Sheep/goat density (heads/km²) | | | Brucellosis incidence (/100000) | | | Correlation statistics | | |
| --- | --- | --- | --- | --- | --- | --- | --- | --- | --- |
|  | 2004 | 2018 | 2020 | 2004 | 2018 | 2020 | r (lag 0–6mo) | P-value | ROC AUC |
| Inner Mongolia | 184 | 258 | 267 | 12.3 | 38.7 | 45.7 | **0.71** | **<0.001** | 0.84 |
| Xinjiang | 98 | 142 | 156 | 8.7 | 19.2 | 23.4 | 0.58 | 0.003 | 0.79 |
| Ningxia | 142 | 175 | 189 | 18.5 | 19.8 | 21.2 | 0.34 | 0.18 | 0.68 |
| Heilongjiang | 45 | 58 | 67 | 2.1 | 3.8 | 4.8 | 0.52 | 0.01 | 0.73 |
| Tibet | 12 | 16 | 19 | 0.4 | 0.7 | 0.9 | 0.41 | 0.09 | 0.65 |
| National Pastoral Average | 96 | 130 | 140 | 5.2 | 12.4 | 14.8 | 0.63 | <0.001 | 0.81 |

Livestock density data from Ministry of Agriculture and Rural Affairs annual censuses. Brucellosis incidence reflects confirmed human cases. Lagged correlation maximized at 6-month moving averages. Inner Mongolia: 23% livestock expansion (2016–2018) with animal vaccination coverage stagnating at 64% (target: 90%). ROC AUC calculated using livestock density threshold >200 heads/km² to predict high-incidence (>30/100k) years.

**Table S8**

Trends in medical service accessibility and rabies case fatality rates in Guangxi between 2008 and 2020

|  |  |  |  |  |  |  |  |  |
| --- | --- | --- | --- | --- | --- | --- | --- | --- |
| Year | Rural healthcare expenditure per Capita (¥) | PEP clinics (n) | Mean distance to PEP clinic (km) | Rabies cases (n) | Rabies deaths (n) | CFR (/1,000) | Correlation (expenditure) | Correlation (PEP clinics) |
| 2008 | 850 | 89 | 45 | 142 | 138 | 971.8 | r = −0.81 | r = −0.74 |
| 2009 | 980 | 102 | 42 | 128 | 124 | 968.8 | P < 0.001 | P < 0.001 |
| 2010 | 1,120 | 124 | 38 | 118 | 113 | 957.6 | (95% CI: −0.92 to −0.58) | (95% CI: −0.88 to −0.47) |
| 2011 | 1,290 | 138 | 35 | 104 | 98 | 942.3 |  |  |
| 2012 | 1,450 | 156 | 32 | 87 | 81 | 931.0 |  |  |
| 2013 | 1,620 | 172 | 29 | 78 | 71 | 910.3 |  |  |
| 2014 | 1,780 | 189 | 26 | 65 | 58 | 892.3 |  |  |
| 2015 | 1,920 | 201 | 23 | 56 | 49 | 875.0 |  |  |
| 2016 | 2,050 | 212 | 21 | 48 | 41 | 854.2 |  |  |
| 2017 | 2,190 | 221 | 20 | 41 | 34 | 829.3 |  |  |
| 2018 | 2,240 | 228 | 19 | 34 | 27 | 794.1 |  |  |
| 2019 | 2,290 | 231 | 18 | 30 | 23 | 766.7 |  |  |
| 2020 | 2,340 | 234 | 18 | 28 | 21 | 750.0 |  |  |

Notes: Correlation coefficients represent time-series associations across all 13 years (2008–2020, n=13 observations). The r and P values are identical for all rows as they describe the overall relationship, not year-specific values. The 95% confidence intervals are provided in the third row for reference.

Data Sources: Rural healthcare expenditure from National Bureau of Statistics; PEP clinic data from Guangxi CDC GIS database; rabies case/death data from CISDCP.

**Table S9**

Correlation of healthcare system capacity with temporal variation in Avian Influenza CFR in Guizhou Province

| Indicator | Guizhou 2010 | Guizhou 2020 | National average 2020 | Guizhou vs. National (%) | Correlation with CFR (r) |
| --- | --- | --- | --- | --- | --- |
| ICU beds per 10,000 population | 1.2 | 2.4 | 4.5 | 53% below | −0.68 (P = 0.03) |
| Mechanical ventilators per 100,000 | 2.8 | 5.1 | 8.2 | 38% below | −0.72 (P = 0.02) |
| Mean time to oseltamivir initiation (hours) | 48 | 24 | 12 | 2× slower | +0.59 (P = 0.05) |
| Laboratory confirmation rate (%) | 58 | 94 | 96 | Similar | −0.12 (P = 0.71) |
| Avian Influenza cases (n) | 8 | 7 | - | - | - |
| Avian Influenza deaths (n) | 6 | 5 | - | - | - |
| CFR (/1,000) | 750.0 | 714.3 | 673.5 (National) | 6.1% higher | - |

Interpretation: Guizhou's persistent high CFR (714.3/1000 vs. 673.5/1000 nationally) aligns with ICU capacity scarcity (53% below national average) and delayed antiviral initiation (24h vs. 12h national average). The stable high CFR despite increased laboratory confirmation (58%→94%) argues against denominator bias as the primary driver.

**Table S10**

ROC analysis performance: rodent density index as predictor of high HFRS incidence years

| Diagnostic Metric | Value | 95% CI | Interpretation |
| --- | --- | --- | --- |
| ROC AUC | **0.73** | 0.61–0.84 | Moderate predictive accuracy (>0.5 = better than chance) |
| Optimal Threshold | Rodent Index >45 | - | Maximizes Youden index (sensitivity + specificity - 1) |
| Sensitivity (True Positive Rate) | 0.78 | - | Correctly identified 78% of high-incidence years |
| Specificity (True Negative Rate) | 0.69 | - | Correctly identified 69% of low-incidence years |
| Positive Predictive Value | 0.70 | - | 70% probability that high rodent index predicts HFRS outbreak |
| Negative Predictive Value | 0.71 | - | 71% probability that low rodent index predicts no outbreak |
| Total Observations | 17 years | 2004-2020 | Based on annual data from Heilongjiang & Inner Mongolia |
| High-Incidence Years (>3/100,000) | 9 years | - | True positives (n=7) + False negatives (n=2) |
| Low-Incidence Years (≤3/100,000) | 8 years | - | True negatives (n=5) + False positives (n=3) |

Data Source: Rodent density index from National Plague Surveillance System (quarterly trapping data, 89 sentinel stations); HFRS incidence from CISDCP confirmed cases.

Clinical Application: A rodent index >45 provides 78% sensitivity and 69% specificity for predicting HFRS high-incidence years, enabling proactive rodent control 6 months before predicted outbreaks (accounting for lag period).

**Table S11**

Sensitivity analysis: Joinpoint APC robustness to surveillance quality and case definition criteria

| Scenario | Disease | Time Period | APC (Original) | APC (Sensitivity) | % Change | P-value | Interpretation |
| --- | --- | --- | --- | --- | --- | --- | --- |
| Scenario 1 Exclude provinces with <80% reporting completeness | Brucellosis | 2004–2014 | +14.2% | +13.8% | −2.8% | <0.001 | Robust |
|  | Malaria | 2004–2020 | −11.5% | −10.9% | −5.2% | <0.001 | Robust |
|  | HFRS | 2004–2008 | −25.6% | −24.1% | −5.9% | <0.001 | Robust |
| Scenario 2 Exclude provinces with lab confirmation <70% | Brucellosis | 2004–2014 | +14.2% | +12.9% | −9.1% | <0.001 | Robust |
|  | Dengue | 2014–2020 | −21.3% | −19.8% | −7.0% | <0.001 | Robust |
|  | Malaria | 2004–2020 | −11.5% | −10.2% | −11.3% | <0.001 | Robust |
| Scenario 3 Restrict to severe diseases (CFR >100/1,000) Rabies, Avian influenza, Plague, HFRS | Overall severe CFR | 2004–2020 | +2.3% | +2.1% | −8.7% | 0.04 | Robust |
|  | Rabies CFR (Guangxi) | 2008–2020 | −9.8% | −9.2% | −6.1% | <0.001 | Robust |
|  | Avian influenza CFR (Guizhou) | 2010–2020 | Stable | Stable | 0% | - | No change |

China CDC reporting completeness scores:

Excluded provinces in Scenario 1: Tibet (65–78% completeness), Qinghai (72–79%), Ningxia (76–81%) for select years.

Lab confirmation rates: Tibet (58–69%), Guizhou (68–72%) pre-2015.

Statistical interpretation: All three scenarios demonstrate that core disease trends remain directionally identical and statistically significant (APC changes <12%, P-values unchanged). This confirms that geographic and temporal conclusions are robust to surveillance heterogeneity and case definition variations, and that the inclusion of rare diseases does not bias main findings.
